# Effect of long-term fertilisation on the weed community of a winter wheat field

**DOI:** 10.1038/s41598-018-22389-4

**Published:** 2018-03-05

**Authors:** Min Jiang, Tao Liu, Niansheng Huang, Xinping Shen, Mingxing Shen, Qigen Dai

**Affiliations:** 1 Innovation Center of Rice Cultivation Technology in Yangtze River Valley, Ministry of Agriculture/Key Laboratory of Crop Genetics and Physiology of Jiangsu Province, Yangzhou University, Yangzhou, 225001 Jiangsu Province China; 2 Lixiahe Agricultural Research Institute of Jiangsu Province, Yangzhou-Jiangsu Collaborative Innovation Center for Modern Crop Production, Yangzhou, China; 3 Agricultural Sciences Research Institute of Taihu Lake District, Yangzhou, China

## Abstract

Effects of fertilisation and other management techniques on a weed community were evaluated during wheat growth in a rice-wheat cropping system. Fertiliser treatments were C0 (C means chemical, C0 means zero chemical fertiliser.), CN (N fertiliser), CNK (N plus K fertiliser), CNPK (N plus P and K fertiliser), CNP (N plus P fertiliser), and CPK (P plus K fertiliser). Weed density, biomass, and bio-diversity were determined. Redundancy analysis (RDA) was used to investigate the relationship between fertiliser management, weed species, and weed density. The overall weed densities in the C0 and CPK treatments were the greatest during wheat seeding and ripening periods and were significantly greater than densities in the other treatments. N, P and organic matter in soil were highly correlated with weed species and density, whereas K in soil was not significantly correlated with weed species and weed density. N fertiliser significantly reduced weed density. Balanced fertilisation maintained weed species richness and resulting in a high yield of wheat. CNPK application reduced weed damage and improved the productivity and stability of the farmland ecosystem.

## Introduction

Weeds are a biological component of all farmland ecosystems. Weed species amount, densities and biomass play important roles in maintaining the biological diversity of farmlands. However, excessive numbers of weeds can compete with crops for water, nutrients and light^1,2^. Fertilisation can alter the natural succession of the weed community in farmland by directly changing the soil nutrient content, as well as impacting crop biomass and yield^3–5^. The species, density, and biomass of weeds occurring with wheat under long-term fertilisation conditions provide insight into the competition between weeds and wheat for nutrients. Reducing the competitiveness of weeds for nutrients might enable population management through modified fertiliser use.

The soil nutrient content significantly affects the weed density, diversity index, and community structure of farmland^6–8^. Proper nutrient management can improve the competitiveness of crops, decrease weed density and alter the species composition of the weed community^9,10^. Ross’ study found that N application increased the density and dry weight of wild buckwheat and wild oat, there are also studies showed that N application had no significant effect on weed density^11,12^. Different response mechanisms to nutrients can lead to variation in weed community structure. Major and Everaarts found that both inorganic and organic fertilisers significantly influence on the weed community. Fertilisation can reduce weed density, increase species diversity, and increase the evenness of the weed community^13–15^.

According to the current research, NPK and herbicide affect the competition between weeds and wheat. However, the experimental conditions are different in these studies, and the effect of NPK and herbicide is also different, which will influence the competition between weeds and wheat, drawing different conclusions about weed density. The possible reason is that short-term or long-term N application, herbicide or no-herbicide application, or their studies were on certain species of weeds. These studies provide a basis for changes in farmland weed communities under different fertilisation conditions. The basic research hypothesis is that rational fertilisation can increase the competitive advantage of wheat, reduce the density and biomass of weeds, achieve higher wheat yield without affecting the weed diversity, to the benefit of forming a high yield and stable wheat field ecosystem. Based on a 31+ year fertilisation experiment in the Taihu area of China, we studied the structural characteristics of the weed community in a wheat field under a variety of NPK fertiliser applications.

## Results

### Differences in soil nutrients and wheat yields

There were six treatments: no fertilisation (C0), N fertiliser only (CN), N fertiliser and P fertiliser (CNP), N fertiliser and K fertiliser (CNK), P fertiliser and K fertiliser (CPK), and all three fertilisers (CNPK). The experiment was initiated in 1980. The initial values for the soil in the experimental farmland were a pH of 6.8 and a bulk density of 1.26 g·cm^−3^. The basic physical and chemical indicators of the topsoil at a depth of 0–15 cm were as follows: organic matter 24.20 g·kg^−1^, total N 1.43 g·kg^−1^, total P 428.00 mg·kg^−1^, available P 8.40 mg·kg^−1^ (Total P includes organic phosphorus and inorganic phosphorus. While Olsen-p is the result of soil available P determination using sodium bicarbonate extraction method (0.5 mol/L NaHCO_3_, i.e. Olsen method), and Olsen-p can be directly absorbed by crops), available K 127.00 mg·kg^−1^.

There were significant differences in farmland soil nutrients and wheat yields among the different fertiliser treatments (Table 1). For the CN and CNK treatments without P fertilisation, the total P content in the soil was equal to the initial P content. The total P and Olsen P in the soil significantly increased in all of the treatments with P fertilisation. The available K content was reduced compared to initial values, depending on the treatments: available K increased by 18.11% in CPK treatment, but decreased by 6.81% −49.61% in the rest treatments. Wheat yield was highest in the balanced NPK fertilisation treatment. N or P deficiency in the soil significantly decreased the wheat yield but differences in available K content did not significantly affect wheat yield.

**Table 1:** Soil nutrient content and wheat yields in different fertilisation treatments.

Treatment	Total N (g·kg^−1^)	Total P (mg·kg^−1^)	Available N (mg·kg^−1^)	Olsen P (mg·kg^−1^)	Available K (mg·kg^−1^)	SOM (g·kg^−1^)	Wheat yield (kg·ha^−1^)
C0	1.54d	341.60c	129.88d	2.20cd	78.00c	28.80d	2,042.00d
CN	1.65bc	368.59c	146.14b	3.83c	71.85cd	28.86bc	3,038.50c
CNP	1.74ab	775.96b	165.33a	15.55b	64.79d	32.00a	4,844.50b
CNK	1.63b	395.93c	159.07ab	1.86d	118.35b	29.04bc	3,493.50c
CNPK	1.80a	757.45b	169.47a	14.42b	77.83c	31.73a	5,527.00a
CPK	1.71abc	923.83a	160.05ab	32.71a	150.39a	30.46ab	2,283.00d

Different lowercase letters n the same column represent significant differences at the 0.05 level.

Total N includes organic N and inorganic N.

Available N are inorganic and include nitrate, and ammonium.

Total P includes organic phosphorus and inorganic phosphorus.

Olsen P is the result of soil available P determination using sodium bicarbonate extraction method.

SOM = soil organic matter.

### Composition of the weed community on the field surface

Seedling and ripening periods are the key periods of wheat growth. According to long-term observation, the weeds in ripening and seedling periods basically covered main weed species of wheat growing periods. And ripening period was the optimum observation time that can directly and obviously looked into the impacts caused by weeds and wheat competition.

During the wheat seeding period (Table 2), the total weed density in the different treatments was (high to low) C0 > CPK > CN > CNK > CNP > CNPK. In addition to the three dominant weed species (*Vicia sativa*, *Lapsana apogonoides*, and *Conyza canadensis*), the dominant weed community contained other species. These included *Ammannia arenara* in the C0 treatment, *Geranium carolinianum*, *Alopecurus aequalis Sobol*, and *Mazus japonicas* in the CNP treatment, *Hemistepta lyrata* and *Alopecurus aequalis Sobol* in the CNPK treatment, and *Mazus japonicas* and *Alopecurus aequalis Sobol* in the CPK treatment. These differences suggest that the fertilisation structure can affect the species composition of the dominant weeds in the early stages of wheat growth. Survey results during the wheat ripening period identified a total of 11 weed species, this was 6 fewer species than the 17 weed species found during the seeding period. The total weed density among the different treatments during the wheat ripening period was (high to low) CPK > C0 > CNK > CN > CNP > CNPK. This order was not consistent with the order observed during the seeding period. The weed species and communities observed during the wheat ripening period differed considerably among the various fertilisation treatments. The dominant weed communities observed during the wheat ripening period significantly differed from those observed during the seeding period. For instance, *Scirpus juncoides* and *Beckmannia syzigachne* were components of the dominant community for C0, CNPK, and CPK treatments during the wheat ripening period but were not in the dominant community during the seeding period. The dry biomass of the weeds increased as weed density increased. Under CPK and C0 treatments dry biomass was significantly greater than in treatments containing N and CPK was significantly greater than C0. Dry weed biomass in the CN, CNPK treatments was significantly lower than in the other treatments, so the N fertilisation and balanced NPK fertilisation appear to be most beneficial for weed reduction.

**Table 2:** Weed species density under different treatments.

	Family	Weed species	C0	CN	CNP	CNK	CNPK	CPK
wheat seeding	Asteraceae	*Gnaphalium affine*	17.67 ± 2.40a	9.00 ± 1.53bc	0.00d	13.00 ± 2.31ab	0.67 ± 0.67d	4.33 ± 0.88cd
		*Lapsana apogonoides*	48.67 ± 2.19b	64.00 ± 3.61a	4.33 ± 0.67c	47.00 ± 3.06b	3.00 ± 0.58c	11.33 ± 2.73c
		*Hemistepta lyrata*	8.33 ± 2.33ab	9.00 ± 1.15ab	4.33 ± 0.33b	15.33 ± 2.03a	11.33 ± 0.33ab	6.00 ± 1.53b
		*Conyza Canadensis*	35.00 ± 2.31a	26.67 ± 2.60ab	18.67 ± 2.33bc	23.67 ± 2.60b	12.33 ± 1.76d	19.67 ± 2.33bc
		*Ixeris denticulate*	3.00 ± 0.58a	1.33 ± 0.67a	1.33 ± 0.88a	2.33 ± 0.33a	3.67 ± 0.67a	2.33 ± 0.33a
	Scrophulariaceae	*Mazus japonicas*	19.67 ± 1.67b	4.00 ± 1.15c	13.00 ± 2.65bc	9.00 ± 1.15bc	4.33 ± 1.45bc	55.00 ± 7.02a
		*Lindernia antipoda*	7.00 ± 2.52a	0.67 ± 0.67a	1.00 ± 1.00a	5.67 ± 1.45a	2.33 ± 0.33a	6.67 ± 1.20a
		*Limnophila sessiliflora*	2.33 ± 0.33abc	4.67 ± 1.20a	2.00 ± 0.00abc	0.67 ± 0.67bc	4.33 ± 1.33ab	0.00c
	Lythraceae	*Ammannia arenara*	56.33 ± 4.33a	7.33 ± 1.45b	3.33 ± 1.33b	8.33 ± 1.45b	3.00 ± 1.00b	11.00 ± 1.53b
		*Ammannia baccifera*	3.00 ± 0.58b	1.33 ± 0.67b	3.33 ± 0.67b	3.33 ± 0.33b	3.00 ± 0.58b	14.67 ± 1.76a
		*Rotala indica*	0.00b	1.33 ± 0.67ab	0.67 ± 0.67ab	0.67 ± 0.67ab	0.67 ± 0.67ab	3.67 ± 1.20a
	Leguminosae	*Vicia sativa*	57.33 ± 4.67a	37.00 ± 3.46b	9.67 ± 2.40c	24.67 ± 2.96bc	8.67 ± 3.18c	74.67 ± 6.96a
	Geraniaceae	*Geranium carolinianum*	18.67 ± 1.20a	16.00 ± 2.08ab	8.67 ± 1.45bc	12.67 ± 2.40ab	3.67 ± 0.88c	8.33 ± 1.45bc
	Gramineae	*Alopecurus aequalis Sobol*	14.67 ± 1.20b	9.00 ± 2.08bc	8.33 ± 1.76bc	5.67 ± 0.88c	13.33 ± 1.33bc	29.00 ± 2.08a
	Caryophyllaceae	*Malachium aquaticum*	1.67 ± 0.88a	0.67 ± 0.67a	0.00a	1.33 ± 0.67a	1.67 ± 0.88a	1.33 ± 0.67a
	Boraginaceae	*Trigonotis peduncularis*	11.33 ± 1.45a	12.33 ± 2.40a	1.33 ± 0.67b	9.67 ± 1.20a	1.33 ± 0.67b	1.33 ± 0.67b
		Group	14.67 ± 0.33a	13.67 ± 0.88a	12.00 ± 0.58a	14.33 ± 0.88a	14.00 ± 0.58a	14.33 ± 0.67a
		Total density	304.67 ± 17.33a	204.33 ± 8.88bc	80.00 ± 9.45d	183.00 ± 6.66c	77.33 ± 5.46d	249.33 ± 15.24b
wheat ripening	Asteraceae	*Lapsana apogonoides*	10.67 ± 4.81a	1.33 ± 1.33a	0.00a	4.00 ± 2.31a	0.00a	1.33 ± 1.33a
		*Conyza Canadensis*	44.00 ± 6.11a	22.67 ± 5.81abc	0.00d	17.33 ± 3.53bcd	2.67 ± 1.33cd	28.00 ± 6.11ab
	Gramineae	*Alopecurus aequalis Sobol*	5.33 ± 1.33c	0.00c	34.67 ± 3.53a	0.00c	17.33 ± 3.53b	5.33 ± 1.33c
		*Beckmannia syzigachne*	4.00 ± 4.00b	1.33 ± 1.33b	6.67 ± 3.53b	0.00b	16.00 ± 10.07b	162.67 ± 7.42a
	Scrophulariaceae	*Mazus japonicas*	34.67 ± 7.42b	8.00 ± 2.31b	21.33 ± 7.42b	8.00 ± 4.00b	8.00 ± 8.00b	136.00 ± 21.17a
	Lythraceae	*Ammannia arenara*	1.33 ± 1.33c	18.67 ± 3.53ab	4.00 ± 4.00c	30.67 ± 3.53a	5.33 ± 1.33bc	13.33 ± 3.53bc
	Leguminosae	*Vicia sativa*	38.67 ± 8.74b	13.33 ± 8.11bc	2.67 ± 2.67c	41.33 ± 5.81b	2.67 ± 1.33c	81.33 ± 9.33a
	Geraniaceae	*Geranium carolinianum*	13.33 ± 3.53b	9.33 ± 3.53b	0.00b	33.33 ± 5.81a	4.00 ± 4.00b	2.67 ± 2.67b
	Umbelliferae	*Daucus carota*	1.33 ± 1.33a	0.00a	0.00a	5.33 ± 3.53a	0.00a	0.00a
	Campanulaceae	*Lobelia chinensis*	12.00 ± 6.93a	0.00a	0.00a	0.00a	5.33 ± 5.33a	14.67 ± 7.42a
	Cyperaceae	*Scirpus juncoides*	38.67 ± 5.81a	4.00 ± 2.31b	0.00b	0.00b	0.00b	1.33 ± 1.33b
		Group	8.67 ± 0.33a	6.00 ± 0.58abc	3.33 ± 0.67c	6.00 ± 0.58abc	5.33 ± 0.33bc	7.67 ± 1.20ab
		Total density	204.00 ± 15.14b	78.67 ± 7.42c	69.33 ± 13.33c	140.00 ± 8.33bc	61.33 ± 12.72c	446.67 ± 49.87a
		Total dry weight	21.29 ± 0.82b	6.17 ± 0.34d	10.02 ± 0.78 cd	13.55 ± 0.44c	8.82 ± 0.54cd	53.02 ± 2.95a

(ind.·m^−2^; g·m^−2^) expressed as the mean ± SE.

Different lowercase letters in the same row represent significant differences at the 0.05 level.

Group refers to the sum of weeds species per treatment.

### Community diversity characteristics of weeds

**Figure 1:**
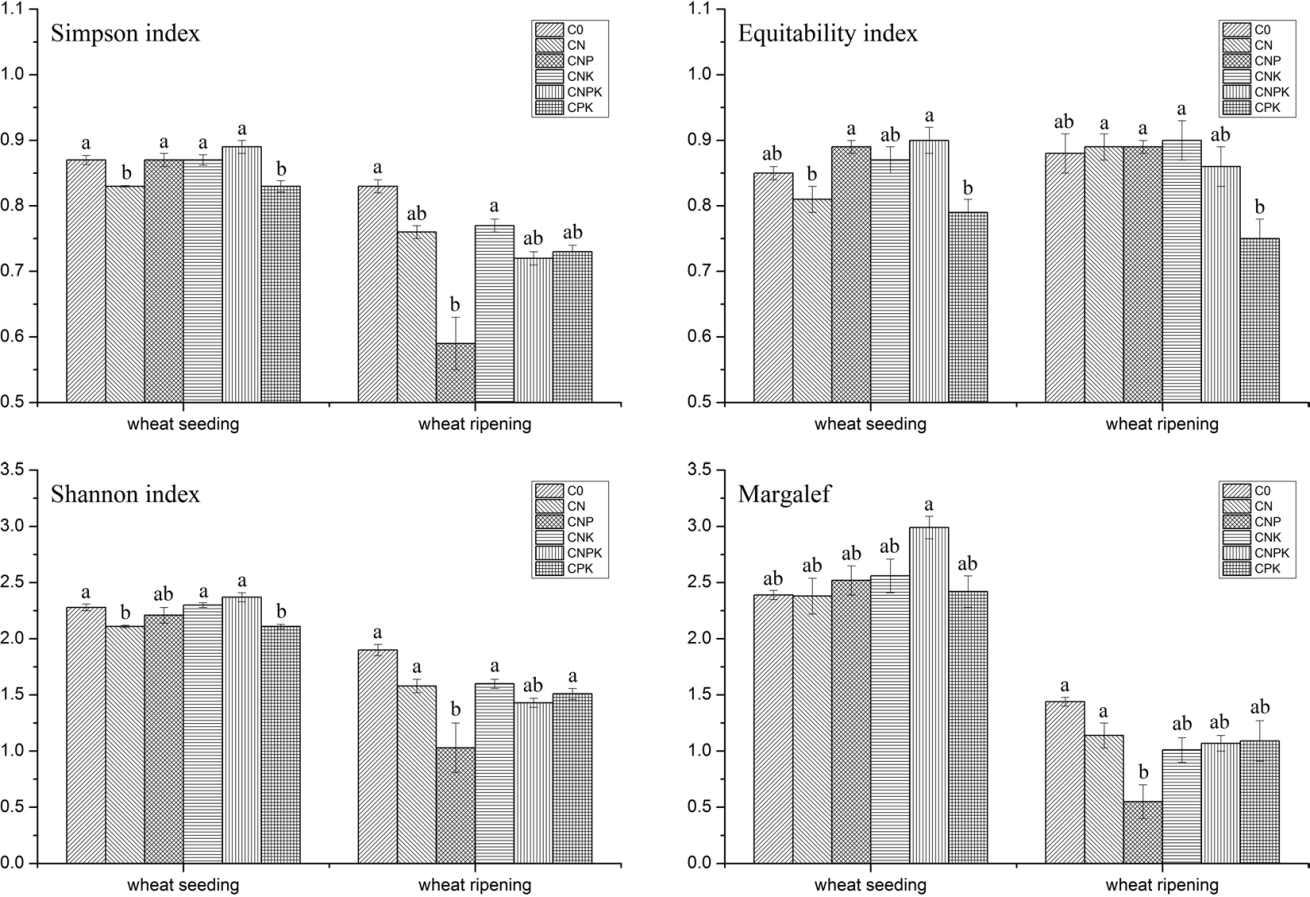
The community structure features under different long-term fertilisation treatments. Different lowercase letters represent significant differences at the 0.05 level. (C0 = no fertiliser application; CN = nitrogen fertiliser only; CNP = nitrogen plus phosphorus fertiliser; CNK = nitrogen plus potassium fertiliser; CPK = phosphorus and potassium fertiliser; CNPK = combined fertiliser with nitrogen, phosphorus, and potassium).

The community diversity of weeds under the long-term treatment with different fertilisers is shown in Fig. 1. The differences in the diversity indexes among the fertilisation treatments were smaller during the seeding period than during the ripening period. In the seedling periods, the CNPK treatment had the highest values and the CPK treatment had the lowest values for all the four diversity indexes. In the ripening periods, C0 treatment had the highest and CNP treatment had the lowest values for Shannon, Simpson and Margalef indexes, while CNK treatment had the highest and CPK treatment had the lowest for Equitability index. The data indicate that the balanced fertiliser application and the no fertiliser treatments increased the diversity of weeds in wheat fields.

### Relationships between weed distribution, soil nutrient factors and wheat yield

**Figure 2:**
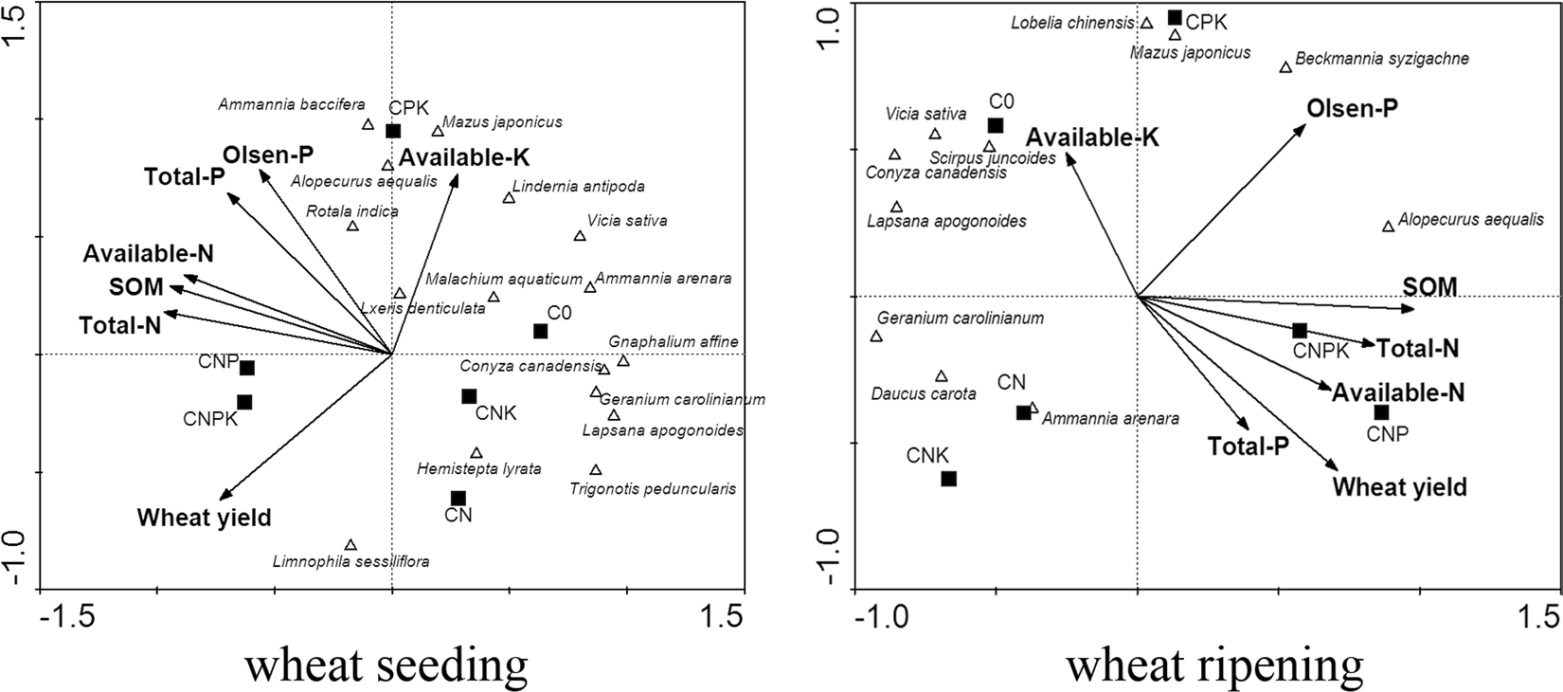
RDA analysis of effects of different fertilisation treatments on weed species and density growth in wheat field. (C0 = no fertiliser application; CN = nitrogen fertiliser only; CNP = nitrogen plus phosphorus fertiliser; CNK = nitrogen plus potassium fertiliser; CPK = phosphorus and potassium fertiliser; CNPK = combined fertiliser with nitrogen, phosphorus, and potassium). Redundancy analysis (RDA), also called reduced-rank regression, the canonical form of PCA. Special cases are simple and multiple regression, analysis of variance and the log-ratio form of reduced-rank regression principal components analysis (PCA). The arrow represents the environmental factor, the quadrant where the arrow is located represents the positive and negative correlation between the environmental factor and the ordination axis, and the length of the arrow connection represents the degree of correlation between an environmental factor and the distribution of the research objects. The longer the connection, the impact of this environmental factor on the distribution of objects is greater. The angle between the arrow connection and the ordination axis represents the correlation between the certain environmental factor and the ordination axis. The smaller the angle is, the higher the correlation is.

Redundancy analysis (RDA) ordination (Table 3, Fig. 2) reflects the general relationship between the weed distribution in the wheat fields and the soil nutrient factors and wheat yields. It also reflects the similarity among weed species for growing requirements.

**Table 3:** The correlation coefficients between the total N, Total P, Available N, Olsen P, SOM, Available K, wheat yield and the first ordination axis.

	Total N	Total P	Available N	Olsen P	SOM	Available K	Wheat yield
wheat seeding period	−0.971	−0.701	−0.884	−0.565	−0.945	0.278	−0.733
wheat ripening period	0.841	0.391	0.687	0.594	0.979	−0.2517	0.708

During the wheat seeding period, the eigenvalues of the first and second ordination axes were 0.560 and 0.282, respectively. During the wheat ripening period, the eigenvalues of the first and second ordination axes were 0.439 and 0.349, respectively. In each ordination, most weeds were located in the opposite direction, opposite the arrow of the soil fertility factor and had a negative correlation with the soil fertility factor, showing a tolerance for unfertile land. Therefore, the application of N and P fertilisers had a significant impact on the weed distribution.

## Discussion

Fertilisation can help to manage weeds by enhancing the competitive advantage of crops^16^. The competition between crops and weeds can be significantly affected by the application of N fertiliser^17^. Many agriculturally important weeds are equally or more responsive than crops to higher soil N levels^18^. Without the use of herbicides, N fertilisation significantly increased weed density and dry matter. The opposite conclusion may be due to the lack of herbicide or short-term N application and other reasons. However, there are fewer articles that are the same as our experimental conditions^19,20^. Our study was based on a 31-year long-term experiment of fertiliser plots. Weeds were planted in conventional high-yielding cultivation of rice and wheat. NPK fertilisers were used in accordance with the growth requirements of wheat, and there will be the use of herbicides during 30–40 days after wheat seedling emergence. Our results indicated that the application of N fertiliser can significantly decrease the weed density in wheat fields. This is likely because N is the primary nutrient factor required to increase crop yield^21,22^. This is likely because, in our study, wheat had higher nitrogen requirements associated to a higher nitrogen uptake rate than the weed species.

Fertiliser application can significantly enhance the NPK content in the soil, alter farmland nutrient habitats, and affect the plant species structure of farmland ecosystems^23^. After P fertilisation, the density of *Mazus japonicas* and *Alopecurus aequalis Sobol* significantly increased, while the density of *Conyza canadensis* considerably decreased. In a 47-year long-term experiment, Banks *et al*. found that *Amaranthus* weeds grew better in fields receiving only P fertiliser. *Mollugo pentaphylla* grew relatively well in fields receiving treatment with only P fertiliser or a NP fertiliser combination, whereas broadleaf weeds did not grow well in fields receiving balanced fertiliser treatments^24^. Different weeds have significantly different demands for various nutrients^25–27^. Katharine *et al*.^28^ demonstrated that a decrease in soil N can increase the intraspecies competition of weeds, whereas P has a greater effect on the interspecies competition. The differences among nutrient levels in the soil caused by fertilisation can determine weed species presence. Therefore, fertilisation can affect the intra- and interspecies competition of farmland weeds, while potentially increasing the competitive advantage of crop plants.

Weeds are important for farmland diversity and changes in agricultural management have reduced the weed diversity. Loreau *et al*.^29^ suggested that the diversity of plant communities is positively correlated with the primary productive forces and is related to soil fertility. Fertilisation can alter the diversity of the weed community in farmland^30,31^. The results of the present study indicate that for the C0 treatment without fertilisation and the CNPK treatment with balanced fertilisation, the individual diversity indexes attained relatively high levels. The wheat yield was also highest with the CNPK treatment. The CPK treatment without N fertilisation, and the CNP treatment, without K fertilisation had relatively low diversity indexes. The CNPK treatment, with balanced fertilisation maintains relatively high diversity and evenness index values and the richness index value was also higher than the other treatments.

Ecological system functioning is influenced by the characteristics and number of species. Differences in the requirements of species for different nutrients result in increased nutrient retention in the system^32^. Proper nutrient management can improve the competitive relationship between crops and weeds. This can reduce the influence of weeds on the crop yield, while maintaining diversity among the controlled weeds. Our RDA results indicated that the N and P nutrient levels in the soil and the organic matter content are the primary environmental factors that affect the distribution of weeds.

Our findings indicated that significant differences in the composition of weeds can be related to fertilisation treatments. N fertiliser had a greater effect on the density of different weed communities, P fertiliser had a greater effect on the weeds species present, and the impact of K fertiliser was unclear. The formation of the weed community in farmland derives from interactions among multiple environmental factors. The combination of NPK fertilisers can significantly reduce the weed density, increase the community diversity index, and achieve high yield and stability of a wheat crop production system.

## Materials and Methods

### Location of the experiment

The long-term experimental farmland study area was located at the Suzhou Academy of Agricultural Sciences in the Taihu area of Jiangsu, China (31°27′45″N, 120°25′57″E). This area has a northern subtropical monsoon climate; annual sunshine is 3,039 h, precipitation is 1,128 mm, average temperature is 15.7 °C, and the effective accumulated temperature is 4,947 °C. The soil used for the experiments was the acidic, heavy, loamy, paddy soil derived from loess sediments.

### Materials for the experiment

The experiment was repeated in triplicate. The area of each small plot was 20 m^2^ (4 m × 5 m), and the plots were permanently separated by ridges using granite slabs and cement, between which the individual small plots were connected with irrigation channels. The crops in this experiment were rice and wheat grown in rotation. Taihu Lake Cultivated Area, encompassing 1.68 million ha, is a high yield rice-wheat region and most fields use a rice-wheat double cropping system. This area is also one of the highest chemical N use areas in China. The total amount of chemical N used in both the rice and the wheat seasons ranges from 500–600 kg ha^−1^.

During the wheat season, the N treatment consisted of urea at 150–300 kg ha^−1^ yr^−1^ (total N) between different years, with approximately 50% as a basal fertiliser, 20% as a tillering fertiliser, and 30% as a wheat earing fertiliser. The P treatment applied P_2_O_5_ at 55.8 kg ha^−1^ yr^−1^, all of which was basal fertiliser. Treatment K applied K_2_O at 137.5 kg ha^−1^ yr^−1^, with 50% as base fertiliser and 50% as wheat earing fertiliser. Chemical weeding was used during both the rice and the wheat seasons. The same herbicides were used in all six treatments. During the rice season, the herbicides were applied 3–5 d after rice transplantation, and consisted of benzyl butyl isoproturon at 750–900 g ha^−1^ and 30% benzyl ethyl pretilachlor wettable powder at 1.5–1.8 L ha^−1^. During the wheat season, 30% benzyl ethyl pretilachlor wettable powder was applied 30–40 d after sowing at 1.5–1.875 L ha^−1^. During herbicide applications in 2011 and 2012, shields were used to block measuring areas to avoid chemical weeding, we inserted shields around each plot with an area of 1 m^2^. Herbicides application was during 30–40 days after wheat seedling. As the wheat grew not high at that time, we used covers to block the test plots when weeding, and after weeding, we removed the covers.

### Survey method

Weed surveys were performed during the wheat seeding and ripening periods of 2011–2012. An iron frame with dimensions of 0.3 m × 0.3 m was used to randomly collect weeds at five sites in each plot. The weed identification was mainly accomplished by referral to the “China’s Farmland Weeds Colored Map”, and identification was verified using China’s Weed Information System. During ripening periods, all the weeds on the field surface were collected and transported to the laboratory. Soil was removed from the weed and they were washed and then dried to a constant weight to determine the total dry matter.

### Soil sampling and nutrient measurement

After the wheat harvest, a soil auger was used to collect soil samples at a 0–15 cm depth from each plot using the diagonal distribution method. The soil collected from the plots was numbered according to the treatment. After being fully mixed, the samples were taken to the laboratory for measurement. The total N in the soil was digested and measured using the Kjeldahl method. The total P was digested with H_2_SO_4_-HC1O_4_ and measured using the molybdenum blue colorimetric method. Available P was determined using a soil extraction followed by colorimetric or inductively coupled plasma atomic emission spectroscopic (ICP-AES) analysis. Olsen-P is one of the most common extractants used, i.e. 0.5 mol/L sodium bicarbonate (NaHCO_3_), which was in our preference due to the soil characteristics and pH of the experimental field. Nascimento *et al*. has reported that analysis of digested samples can in some cases cause the determination of inaccurate P concentration; however, the methods employed in the current manuscript were only methods available^33^. The alkaline hydrolysis N was measured by the alkaline hydrolysis diffusion method. Available P was measured with the sodium bicarbonate method. Available K was measured with the ammonium acetate extraction method. Organic matter was measured by the potassium dichromate volumetric method.

### Data processing and analysis

The species diversity of the weeds was characterized by applying the following indexes.

The species diversity was calculated by the Shannon index: $H\text{'}=-\sum _{i=1}^{s}Pi\,\mathrm{ln}\,Pi$, where *S* is the total number of species in a single treatment. *P*_*i*_ is the species abundance of species *i*, *P*_*i*_ is calculated as ${P}_{i}=\frac{{n}_{i}}{N}$; where *n*_*i*_ is the number of individuals of species *i*, and *N* is the total number of individuals of all of the species in the block.

The community dominance was determined by the Simpson index: $D=1-\sum _{i=1}^{s}{P}_{i}^{2}$.

The community evenness was calculated by the Pielou index of evenness: $J=\frac{H\text{'}}{\mathrm{ln}\,S}$.

Species richness was determined using the Margalef index: $R=\frac{S-1}{\mathrm{ln}\,N}$.

Simpson index and Shannon index are aggregative indicators to evaluate species diversity and individual distribution evenness. Shannon index brings out the uncertainty of the community, bases on the species number of weed community. The value of this index is larger, the samples have better evenness. Simpson index refers to the probability that two individuals randomly selected from a sample will belong to different species. Ranging from 0 to 1, the higher the index, the higher the diversity.

Margalef index refers to the species richness degree in a community or environment. The number of species per sample is a measure of richness. The more species present in a sample, the ‘richer’ the sample. Pielou index intends to estimate the evenness rate of weed species distribution. Evenness is a measure of the relative abundance of the different species. Pielou bases on Shannon index (H′) and biggest diversity value (Hmax), ranges from 0 to 1.

The basic data processing and statistical analyses were performed using Excel 2003 and SPSS 16.0, and the redundancy analyses of the soil nutrient data and the weed density data were performed using Canoco 4.5. The figures were drawn using Origin 8.5.

## Electronic supplementary material

Supplementary information - Photos of Shields
