# Supplementary material for: Effect of long-term fertilisation on the weed community of a winter wheat field
Source: Sci Rep. 2018 Mar 5;8:4017. doi: 10.1038/s41598-018-22389-4 (PMC5838251; doi:10.1038/s41598-018-22389-4)

Effect of long-term fertilisation on the weed community of a winter wheat field

Min Jiang[1,2**], Tao Liu[1**], Niansheng Huang[2], Xinping Shen[1], Mingxing Shen[3], Qigen Dai[1*]

Supplementary Information

Photos of Shields

We inserted shields around each plot with an area of 1m². Herbicides application was during 30-40 days after wheat seedling. As the wheat grew not high at that time, we used covers to block the test plots when weeding, and after weeding, we removed the covers.

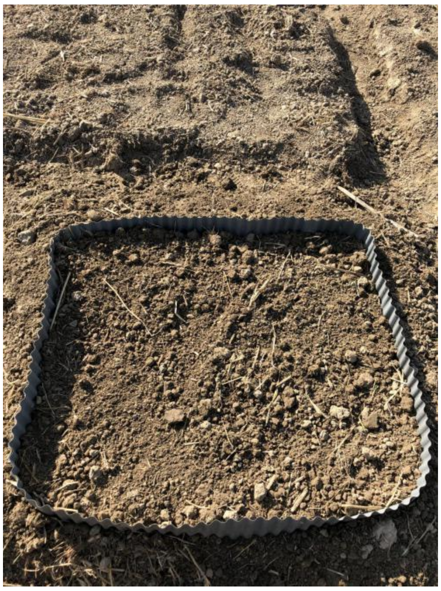

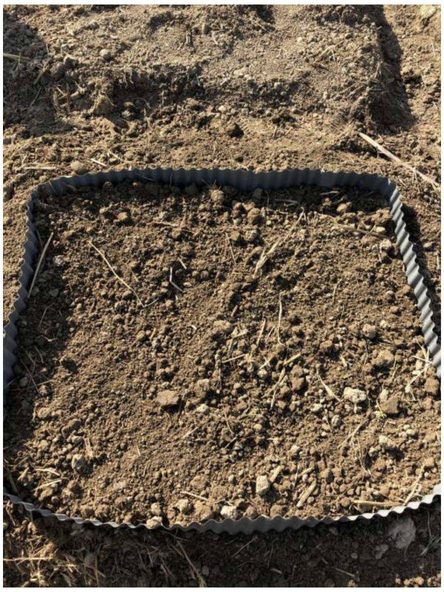

Supplement: Supplementary file 1 — Supplementary information - Photos of Shields [file 41598_2018_22389_MOESM1_ESM.pdf]
